# Epidemiologic relationship between periodontitis and type 2 diabetes mellitus

**DOI:** 10.1186/s12903-020-01180-w

**Published:** 2020-07-11

**Authors:** Chen-zhou Wu, Yi-hang Yuan, Hang-hang Liu, Shen-sui Li, Bo-wen Zhang, Wen Chen, Zi-jian An, Si-yu Chen, Yong-zhi Wu, Bo Han, Chun-jie Li, Long-jiang Li

**Affiliations:** 1 State Key Laboratory of Oral Diseases & National Clinical Research Center for Oral Diseases & Department of Head and Neck Oncology, West China Hospital of Stomatology, Sichuan University, Number 14, Unit 3, Renmin Nan Road, Chengdu City, 610041 Sichuan China; 2 State Key Laboratory of Oral Diseases & National Clinical Research Center for Oral Diseases & Department of Orthognathic and Temporomandibular Joint Surgery, West China Hospital of Stomatology, Sichuan University, Chengdu, China

**Keywords:** Diabetes mellitus, type 2, Periodontitis, Systematic review, Meta-analysis, Epidemiologic studies

## Abstract

**Background:**

To systematically review the epidemiologic relationship between periodontitis and type 2 diabetes mellitus (T2DM).

**Methods:**

Four electronic databases were searched up until December 2018. The manual search included the reference lists of the included studies and relevant journals. Observational studies evaluating the relationship between T2DM and periodontitis were included**.** Meta-analyses were conducted using STATA.

**Results:**

A total of 53 observational studies were included. The Adjusted T2DM prevalence was significantly higher in periodontitis patients (OR = 4.04, *p* = 0.000), and vice versa (OR = 1.58, p = 0.000). T2DM patients had significantly worse periodontal status, as reflected in a 0.61 mm deeper periodontal pocket, a 0.89 mm higher attachment loss and approximately 2 more lost teeth (all *p* = 0.000), than those without T2DM. The results of the cohort studies found that T2DM could elevate the risk of developing periodontitis by 34% (*p* = 0.002). The glycemic control of T2DM patients might result in different periodontitis outcomes. Severe periodontitis increased the incidence of T2DM by 53% (*p* = 0.000), and this result was stable. In contrast, the impact of mild periodontitis on T2DM incidence (RR = 1.28, *p* = 0.007) was less robust.

**Conclusions:**

There is an evident bidirectional relationship between T2DM and periodontitis. Further well-designed cohort studies are needed to confirm this finding. Our results suggest that both dentists and physicians need to be aware of the strong connection between periodontitis and T2DM. Controlling these two diseases might help prevent each other’s incidence.

## Background

Diabetes mellitus (DM) is a common metabolic disease resulting from a defect in insulin secretion, a defect in insulin action or a combination of both [1]. Type 2 DM (T2DM) results from the body’s ineffective use of insulin and comprises 90% of people with DM worldwide [2]. The number of people with DM has risen rapidly in the last several decades from 108 million in 1980 to 422 million in 2014, and the number is likely to be more than double in the next 20 years. Furthermore, the WHO projected that diabetes will be the seventh leading cause of death in 2030 [3].

Periodontitis is a chronic, multifactorial inflammatory disease in the underlying supporting tissues surrounding the teeth. Sufferers may experience gingivitis, loss of periodontal attachment, resorption of alveolar bone, and eventually tooth loss [4]. Severe periodontitis, which is the sixth most prevalent chronic disease among the general population, affects nearly 750 million people worldwide and is thought to affect people’s chewing ability, nutritional status and quality of life [5, 6].

T2DM and periodontitis have a bidirectional relationship that is well documented in many reviews and epidemiological studies [7–9]. Periodontitis is defined as the sixth complication of DM, which means that DM can promote the progression of periodontitis [10]. Conversely, periodontitis is now known as a risk factor for worsening glycemic control and may increase the risk for diabetic complications [11]. Mechanistically, T2DM influences periodontitis initiation and progression by causing a hyperinflammatory response, impairing bone repair processes, and producing advanced glycation end products [9, 12, 13]. Periodontitis as a local focus of infection can cause the levels of IL-6, TNF-a, and CRP to increase in systems, resulting in increased systemic inflammation, which contributes to insulin resistance [14]. Based on the biological hypothesis, there are substantial randomized controlled trials (RCTs) that show periodontal treatment can improve glycemic control [15]. However, two well-designed large-scale RCTs obtained contradictory results on whether periodontal treatment had an effect on glycated hemoglobin (HbA1c) in T2DM patients [16, 17].

The above contradiction raised our curiosity. Do these two common diseases truly affect each other? However, after systematically searching the literature, we found that there was no systemic review to date that answers this question comprehensively. In the present work, we summarized evidence from observational studies to explore this bidirectional relationship.

## Methods

The protocol of the present systematic review was registered in PROSPERO (CRD42018089993). All procedures were performed following this protocol and in accordance with the MOOSE statements [18]. Two authors independently achieved study selection, quality assessment and data extraction. Any controversies were solved by consensus discussion.

### Search strategy

The search strategy was a combination of an electronic search and a manual search. The manual search included the reference lists of the included studies and the following journals: Diabetes Care, Journal of Periodontology, Journal of Clinical Periodontology and Journal of Dental Research. The following electronic databases were searched without language limitations: MEDLINE (OVID, 1948 to December 2018), EMBASE (OVID, 1984 to December 2018), Chinese BioMedical Literature Database (CBM, 1978 to December 2018), and China National Knowledge Infrastructure (CNKI, 1994 to December 2018). MeSH terms with free text words were combined when conducting electronic searches. The MeSH terms used for periodontitis were “periodontal diseases” and “periodontitis”. The free text word was “(periodont$ or gingivitis or gingiva$ or gum$).mp.”. The MeSH term used for T2DM searching was “diabetes mellitus, type 2”. Free text words were “(((non-insulin or noninsulin or type 2 or type II or matur$ or adult) adj4 (DM or diabet$)) or T2DM or DMT2 or NIDDM or MODY).mp.”. The titles and abstracts were initially scanned, and the full texts of the possibly eligible studies were obtained for final judgment.

### Inclusion criteria

Observational studies (cross-sectional studies, case-control studies and cohort studies) investigating the relationship between T2DM and PD were included. The criteria for the outcomes for periodontitis were clinical attachment loss (CAL), periodontal pocket depth (PPD), number of teeth (NOT), loss of teeth (LOT), alveolar bone loss and community periodontal index (CPI) score. The criteria for the outcomes for T2DM were oral glucose tolerance test (OGTT), HbA1c and fasting plasma glucose (FBG) results. Disease (periodontitis or T2DM) prevalence and incidence were also included. The participants chosen represent the natural population grouping into periodontitis versus non-periodontitis or T2DM versus non-DM. Comparisons based on periodontitis parameters, such as the T2DM incidence/prevalence between patients with low CAL levels and high CAL levels, were also included. Studies investigating outcomes in selected populations, such as comorbid patients, all periodontitis patients, all T2DM patients or all healthy participants (periodontitis-free and T2DM-free), were excluded. Studies were selected according to the aforementioned periodontitis/T2DM-related parameters, medical records or self-reported medical history.

### Methodological quality assessment

The study quality of cohort studies and case-control studies were measured by the Newcastle-Ottawa Scale (NOS) scoring system. Studies with scores less than 3 were regarded as low quality and were excluded. For cross-sectional studies, the Agency for Healthcare Research and Quality (AHRQ) scoring system was applied. Studies with scores less than 3 in the AHRQ scoring system were regarded as low quality and were not included.

### Data extraction

The extracted data were as follows: 1) investigator, 2) country, 3) number of participants, 4) age and sex of the participants, 5) recruitment of participants, 6) selected outcomes, and 7) NOS/AHRQ score. For cohort studies, the follow-up period and number of incident cases were also extracted.

### Data analysis

The software STATA 14.0 was utilized for meta-analysis. Weighted mean differences (WMDs) with 95% confidence intervals (CIs) were calculated for continuous data. Odds ratios (ORs) and risk ratios (RRs) with 95% CIs were calculated for dichotomous data. Generic inverse variance (lnOR or lnRR) was used for meta-analyses that included studies that only reported ORs or RRs. Significance was determined by two-sided α values with a cut-off *p* value of 0.05. All meta-analyses were performed under the random-effects model. Cochran’s Q test and I^2^ statistic were used to detect statistical heterogeneity among studies. When *P* > 0.10 and I^2^ < 50%, the study was regarded as having low heterogeneity; otherwise, it was regarded as having high heterogeneity. Meta-regression was utilized for a meta-analysis that included more than 4 studies to investigate possible sources of heterogeneity. The influence test was conducted by deleting every single study in turn to test whether the results were stable. For a meta-analysis that included more than 10 studies, publication bias was detected by Egger’s test and Begg’s test. The publication was excluded when both test results exhibited *p* > 0.05. If publication bias existed or unstable results were found, the trim and fill method was applied.

## Results

### Results of the search and characteristics of the included studies

**Fig. 1:**
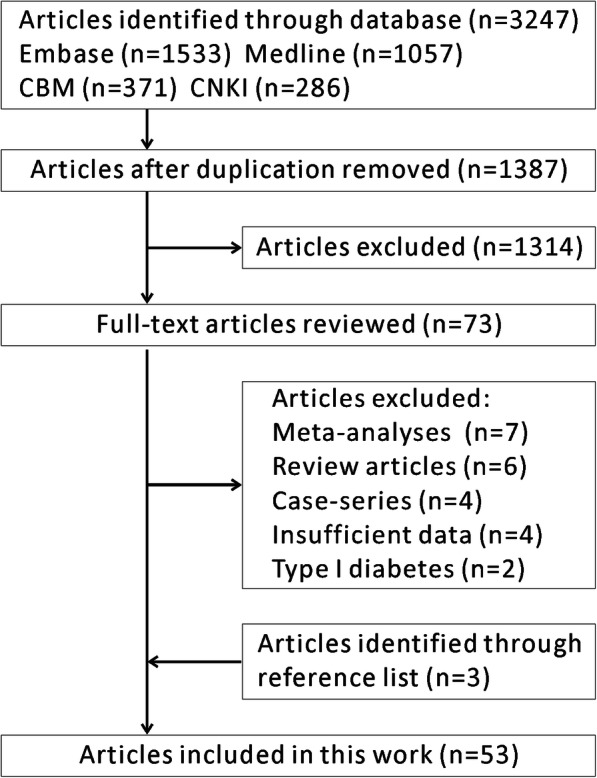
Flow chart of the study selection

A total of 1387 studies were identified from the primary search after removing duplicate studies. After screening the titles and abstracts, 73 studies were identified for further evaluation. After browsing the full text, 50 studies were considered eligible for inclusion. Twenty-three studies were excluded for various reasons. Among these, 16 studies were excluded because of the study type (7 meta-analyses, 6 review articles and 3 case series reporting periodontal treatment for T2DM patients); 4 studies were excluded because the reported outcomes were insufficient; 2 studies were excluded because they included type 1 DM. Additional reference checking revealed 3 studies that were then included. Journal searching did not add any new studies. Finally, a total of 53 studies were included in the present work. Figure 1 shows the search and inclusion process. Appendix Tables S1 and S2 summarize the characteristics of 43 [19–61] cross-sectional studies and 12 [23, 39, 62–71] cohort studies, respectively. Appendix Tables S3 and S4 summarize the AHRQ and NOS scores of cross-sectional studies and cohort studies. All included cross-sectional studies and cohort studies had scored higher than 3.

After systematically reviewing the included studies, we found that they answered 3 questions (questions 1–3, Q1–3). Specifically, cross-sectional studies gave the answer “Q1: Are periodontitis and T2DM associated with each other?” Cohort studies gave the answer to the other two questions: “Q2: Does T2DM increase the risk of developing periodontitis?”, and “Q3: Does periodontitis increase the risk of developing T2DM?”

## Results of meta-analyses

### Q1: are periodontitis and T2DM associated with each other?

A total of 43 cross-sectional studies were included to answer Q1. Evidence was from some national large-scale population-based studies, such as the SHIP, NHANES and KCIS, and some small-sample studies recruiting participants from communities or hospitals. Among these studies, only 14 studies reported adjusted outcomes (Table 1). Six meta-analyses were conducted as follows.

**Table 1:** Summary of adjusted results of cross-sectional studies

Study	Evaluated PD related conditions	Definition of T2DM	Main conclusion and outcome
**PD/non-PD**			
Awuti 2012 [20]	Moderate PD: PPD ≤6 mm, or CAL of 3–4 mm; or possible presence of slight loose teeth (*N* = 98) Severe PD: PPD > 6 mm, or CAL ≥5 mm; or more than one loose tooth (*N* = 77) Control: non-PD (*N* = 509)	The 1999 WHO criteria and ADA standards	T2DM was more prevalent in moderate PD compared with no PD. Adjusted OR = 4.033, 95%CI 2.069–7.861 T2DM was more prevalent in severe PD compared with no PD. Adjusted OR = 2.313, 95%CI 1.042–5.137
Choi 2011 [22]	Top quintile category versus the bottom quintile CAL: Quintile 1 mean CAL = 0.2 mm (*N* = 2412) Quintile 5 mean CAL = 3.0 mm (*N* = 2453)	ADA criteria	T2DM was more prevalent in mean CAL 3.0 mm compared with mean CAL 0.2 mm. Adjusted OR = 4.77, 95%CI 2.69–8.46
	Top quintile category versus the bottom quintile PPD: Quintile 1 mean PPD = 0.7 mm (*N* = 2451) Quintile 5 mean PPD = 2.2 mm (*N* = 2449)		T2DM was more prevalent in mean PPD 2.2 mm compared with mean PPD 0.7 mm. Adjusted OR = 1.63, 95%CI 1.10–2.42
Mohamed 2013 [37]	Chronic PD: at least one site with PPD of > 4 mm (*N* = 290) Control: non-PD (*N* = 157)	The 1999 WHO criteria	T2DM was more prevalent in chronic PD compared with non-PD. Adjusted OR = 4.07, 95%CI 1.74–9.49
	Tooth mobility (*N* = 153) Control: without tooth mobility (*N* = 294)		T2DM was more prevalent in participants with tooth mobility compared with those without. Adjusted OR = 5.90, 95%CI 2.26–15.39
	NOT > 21 teeth (*N* = 381) Control: NOT≤21 teeth (*N* = 66)		T2DM was less prevalent in participants with > 21teeth, with an OR of 0.23. Adjusted OR = 0.23, 95%CI 0.08–0.63
Nesse 2010 [40]	PD: CPITN score was ≥3, indicating PPD ≥4 mm (*N* = 217) Control:non-PD (*N* = 320)	Clinical examination; or medical record	T2DM was more prevalent in PD compared with non-PD. Adjusted OR = 4, 95%CI 1.03–15.3
Saito 2004 [46]	high portion category compared in the low portion CAL: Low mean CAL < 1.5 mm (N = 18) High mean CAL > 2.5 mm (*N* = 38)	The WHO criteria	T2DM was more prevalent in mean CAL > 2.5 mm compared with mean CAL 0.2 mm. Adjusted OR = 2.0, 95%CI 1.0–3.9
	PPD: Low mean PPD < 1.3 mm (*N* = 18) High mean PPD > 2.0 mm (*N* = 32)		T2DM was more prevalent in mean PPD > 2.0 mm compared with < 1.3 mm. Adjusted OR = 2.6, 95%CI 1.3–5.0
Saito 2006 [45]	Mean alveolar bone loss (*N* = 131) Control: Low alveolar bone loss (N = 49)	The WHO criteria	Mean alveolar bone loss as a continuous variable showed a 1% increase in mean alveolar bone loss corresponded to a 6% increased prevalence of T2DM. Adjusted OR = 1.06 95%CI 1.00–1.12
**T2DM/non-T2DM**			
Kaur 2009 [25]	Top quartile compared with three lower quartiles LOT (Quartile 4 vs 1–3)	T2DM: After the age of 29; or insulin started > 1 year after disease onset (*N* = 310) Non-T2DM (*N* = 1858)	The OR for increase tooth loss was 1.65 times higher for the T2DM patients compared with non-T2DM participants. Adjusted OR = 1.65, 95%CI 1.13–2.39
Kowall 2015 [27]	PD: at least 2 non-adjacent teeth CAL ≥ 3 mm	Poorly controlled T2DM:HbA1c ≥7% (*N* = 64) Better controlled T2DM:HbA1c < 7% (*N* = 137) Non-T2DM (*N* = 2145)	PD was more prevalent in poorly controlled T2DM patients compared with non-T2DM participants, which was not statistically significant. Adjusted OR = 1.60 95%CI 0.55–4.63 The prevalence of PD showed no difference between better controlled T2DM patients and non-T2DM participants. Adjusted OR = 0.94 95%CI 0.52–1.67
	Top quartile compared with three lower quartiles Mean CAL ≥ 4 mm (Quartile 4 vs 1–3)		The OR for CAL ≥ 4 mm was 1.36 times higher in poorly controlled T2DM patients compared with non-T2DM participants, which was not statistically significant. Adjusted OR = 1.36 95%CI 0.75–2.49 The prevalence of CAL ≥ 4 mm showed no difference between better controlled T2DM patients and non-T2DM participants. Adjusted OR = 0.94 95%CI 0.61–1.45
	Top quartile compared with three lower quartiles Mean PPD (Quartile 4 vs 1–3)		The OR for top PPD was 1.31 times higher for the poorly controlled T2DM patients compared with non-T2DM participants, which was not statistically significant. Adjusted OR = 1.31 95%CI 0.75–2.30 The prevalence of mean PPD showed no difference between better controlled T2DM patients and non-T2DM participants. Adjusted OR = 1.13 95%CI 0.75–1.71
	Lowest quartile compared with three higher quartiles NOT (Quartile1 vs 2–4)		The OR for NOT was 1.49 times higher in poorly controlled T2DM patients compared with non-T2DM participants, which was no statistically significant Adjusted OR = 1.49 95%CI 0.92–2.40 NOT showed no difference between better controlled T2DM patients and non-T2DM participants. Adjusted OR = 1.05 95%CI 0.74–1.50
Leung 2008 [30]	Chronic PD: CPI score of 4 in any one sextant (WHO, 1997).	T2DM: Clinical examination; or medical record (*N* = 364) Non-T2DM (*N* = 161)	PD was more prevalent in T2DM patients compared with non-T2DM participants. Adjusted OR = 1.84 95%CI 1.22–2.77
	CAL ≥ 6 mm		The OR for CAL ≥ 6 mm was 1.71 times higher for T2DM patients compared with non-T2DM participants. Adjusted OR = 1.71, 95%CI 1.13–2.59
Nelson 1990 [39]	PD: < 24 teeth present;> 6 teeth with ≥25% bone loss and any tooth with ≥50% bone loss.	T2DM: OGTT ≥11.1 mmol/l (*N* = 720) Non-T2DM (*N* = 1553)	PD was more prevalent in T2DM patients compared with non-T2DM patients. Adjusted OR = 1.64, 95%CI 1.50–1.79
Saito 2005 [47]	Mean PPD ≥1.9 mm	T2DM: The WHO criteria (*N* = 27) Non-T2DM (*N* = 360)	The OR for PPD ≥ 1.9 mm was 1.4 times higher for the T2DM patients compared with non-T2DM participants, which was not statistically significant. Adjusted OR = 1.4 95%CI 0.6–3.2
	Mean CAL ≥2.42 mm		The OR for CAL ≥ 2.42 mm was 1.5 times higher for the T2DM patients compared with non-T2DM participants, which was not statistically significant. Adjusted OR = 1.5 95%CI 0.7–3.2
Tanwir 2009 [51]	Missing fewer teeth	T2DM: Clinical examination; or medical record (*N* = 88) Non-T2DM (*N* = 80)	The OR for missing or fewer teeth was 2.3 times higher for the diabetic patients compared with non-T2DM patients. Adjusted OR = 2.3 95%CI 1.32–4.14
Tsai 2002 [52]	Severe PD: at least two sites CAL ≥6 mm at least one site PPD ≥5 mm	Poorly control T2DM:HbA1c ≥9% (*N* = 170) Better control T2DM:HbA1c < 9% (*N* = 260) Non-T2DM (*N* = 3841)	Severe PD was more prevalent in poorly controlled T2DM patients compared with non-T2DM participants. Adjusted OR = 2.90 95%CI 1.40–6.03 Severe PD was more prevalent in better controlled T2DM patients compared with non-T2DM participants, but was not statistically significant. Adjusted OR = 1.56 95%CI 0.90–2.68
Wang 2009 [53]	PD: The WHO 1997 criteria	T2DM: The 1999 WHO criteria (*N* = 193) Non-T2DM (*N* = 8468)	PD was more prevalent in T2DM patients compared with non-T2DM participants. Adjusted OR = 1.34 95%CI 1.07–1.74

PD: periodontitis; T2DM: type 2 diabetes mellitus; CAL: clinical attachment loss; PPD: periodontal pocket depth; NOT: number of teeth; LOT: loss of teeth; HbA1c: glycated hemoglobin; OR: odds ratio; CPI: community periodontal index; RPI: Russell periodontal index

### Strength of association between periodontitis and T2DM

A total of 15 cross-sectional studies with 17,924 participants reported the unadjusted OR between these two diseases (Table S1). Since the original data were not directionally adjusted, a meta-analysis was not undertaken. Among the 15 studies, except for 4 studies [21, 34, 43, 51] that reported that the presence of periodontitis was not different between T2DM patients and non-T2DM controls, all the other studies acknowledged that there was a strong connection.

### Directional adjusted T2DM prevalence (periodontitis versus nonperiodontitis)

**Fig. 2:**
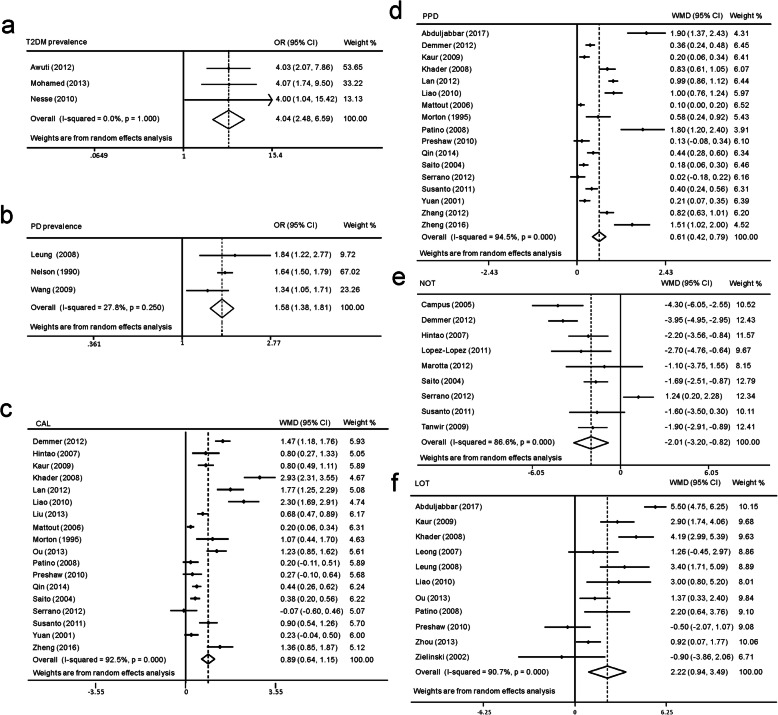
Meta-analyses of cross-sectional studies. (**a**) Results of adjusted ORs on T2DM prevalence (**b**) Results of adjusted ORs on periodontitis prevalence (**c**) Results of crude CAL (**d**) Results of crude PPD (**e**) Results of crude NOT (**f**) Results of crude LOT

A total of 6 cross-sectional studies were included, and all had T2DM prevalence as an outcome. Three studies with 1956 participants were included in a meta-analysis that included a periodontitis diagnosis as an outcome. The included studies had no significant heterogeneity. The results showed that periodontitis patients had significantly higher odds of T2DM prevalence than participants with no periodontitis (OR = 4.04, 95% CI 2.48–6.59, *p* = 0.000, Fig. 2a). Influence analysis showed that the pooled result was stable (Fig. S1a). Other exposure factors included CAL, PPD, LOT, tooth mobility and alveolar bone loss. The results all proved that T2DM was more prevalent in participants with worse periodontal health (Table 1).

### Directional adjusted periodontitis prevalence (T2DM versus non-DM)

A total of 8 cross-sectional studies were included, and all took T2DM as exposure. Three studies with 11,459 participants were included in a meta-analysis evaluating periodontitis prevalence. No significant heterogeneity was detected. The results showed that T2DM patients had a significantly higher ORs for PD prevalence (OR = 1.58, 95% CI 1.38–1.81, *p* = 0.000, Fig. 2b). Influence analysis indicated that the pooled result was stable (Fig. S1b). In addition to periodontitis prevalence, other outcomes were divergent. In brief, all studies demonstrated that periodontitis-related parameters were more prevalent in T2DM patients, although some of the differences were not statistically significant. The results are summarized in Table 1.

### CAL level differences between T2DM and DM-free participants

Eighteen cross-sectional studies with 9571 participants were included. Significant heterogeneity was detected (*p* = 0.000; I^2^ = 92.5%). Pooled results showed that T2DM patients had a 0.89 mm higher CAL than controls (WMD = 0.89, 95% CI 0.64–1.15, p = 0.000, Fig. 2c). Influence analysis demonstrated that the pooled result was stable (Fig. S1c). Publication bias was detected by Egger’s and Begg’s tests (Egger, *p* = 0.003; Begg, *p* = 0.015). Then, we employed the trim and fill method to further evaluate publication bias and found that the results were still significantly positive after adding the hypothesized studies (Table S5).

### PPD differences between T2DM and DM-free participants

Seventeen cross-sectional studies with 8982 participants were included. Significant heterogeneity was detected (*P* = 0.000; I^2^ = 94.5%). Pooled results showed that the periodontal pockets of T2DM patients were 0.61 mm deeper than those of controls (WMD = 0.61, 95% CI 0.42–0.79, *p* = 0.000, Fig. 2d). Influence analysis demonstrated that the pooled result was stable (Fig. S1d). Publication bias was detected by Egger’s and Begg’s test (Egger, *p* = 0.015; Begg, *p* = 0.006). However, adding hypothesized studies by the trim and fill method still resulted in strong significance (Table S5).

### NOT differences between T2DM and DM-free participants

Nine cross-sectional studies with 4415 participants were included. Significant heterogeneity was detected (*p* = 0.000; I^2^ = 86.6%). Pooled results showed that T2DM patients had, on average, 2.01 fewer teeth remaining than controls. (WMD = -2.01, 95% CI -3.20--0.82, p = 0.000, Fig. 2e). Influence analysis demonstrated that the pooled result was stable (Fig. S1e). No publication bias was detected (Egger, *p* = 0.723; Begg, *p* = 0.917).

### LOT differences between T2DM and DM-free participants

Eleven cross-sectional studies with 3405 participants were included. Significant heterogeneity was detected (*P* = 0.000; I^2^ = 90.7%). Pooled results showed that T2DM patients had, on average, lost 2.22 more teeth than controls (WMD = 2.22, 95% CI 0.94–3.49, *p* = 0.000, Fig. 2f). Influence analysis demonstrated that the pooled result was stable (Fig. S1f). No publication bias was detected (Egger, *p* = 0.230; Begg, *p* = 0.755).

### Meta-regression for meta-analyses with huge heterogeneity

Huge statistical heterogeneity existed in the above 4 meta-analyses, and the I^2^ ranged from 86.3 to 94.5%; thus, we performed meta-regression to find the possible sources of heterogeneity. The available covariates included the number of participants, mean age, sex composition of the participants, geographic area and AHRQ scores. However, single variable regression did not find any significant covariates; multiple regression of these covariates only explained approximately 10% of the heterogeneity of all meta-analyses (data not shown). The significant heterogeneity might be caused by statistical heterogeneity or other potential clinical diversity not included in the meta-regression.

#### Q2: does T2DM increase the risk of developing periodontitis?

A total of 6 cohort studies were considered eligible. The results are summarized in Table 2. Two meta-analyses on periodontitis incidence were performed as follows. In addition to periodontitis incidence, other outcomes, including LOT, PPD, CAL and alveolar bone loss, were also reported. The results are summarized in Table 2.

**Table 2:** Summary of results of cohort studies

Study	Characteristics	Definition of outcome	Definition of exposure	Main conclusion and outcome
**T2DM/non-T2DM**				
Chiu 2015 [62]	Taiwan, KCIS study 5y FU (2003–2008)	Binary variable PD: CPI ≥ 3 Non-PD: CPI < 3	T2DM: FBG ≥ 126 mg/dl or self-reported T2DM (*N* = 57) Pre-diabetes: 100 ≤ FBG < 126 mg/dl (*N* = 297) None: FBG < 100 mg/dl (*N* = 4033)	T2DM led to a 95% elevated risk for incident PD. Adjusted HR = 1.95, 95%CI 1.22–3.13 Pre-diabetes led to a 25% elevated risk for incident PD. Adjusted HR = 1.25, 95%CI 1.00–1.57
Demmer 2012 [23]	Germany, SHIP study 5y FU (1997–2006)	Binary variable Tooth loss or not	T2DM: Self-reported age > 30 years old, or HbA1c ≥ 6.5%, timing of insulin therapy initiation > 1 year from diagnosis Controlled T2DM: HbA1c ≤ 7% (*N* = 80) Uncontrolled T2DM: HbA1c > 7% (N = 72) Control: no DM (*N* = 2280)	Controlled T2DM did not lead to an elevated risk for tooth loss. Adjusted RR = 1.01, 95%CI 0.79–1.28 Uncontrolled T2DM led to a 36% elevated risk for tooth loss. Adjusted RR = 1.36, 95%CI 1.11–1.67
		Continuous variable Mean PPD change; Mean CAL change;		Controlled T2DM did not lead to an increased PPD and CAL change. Adjusted MD = 0.04 and 0.09 mm, *p* > 0.05 Uncontrolled T2DM led to a significant increase in PPD and CAL change. Adjusted MD = 0.18 and 0.37 mm, *p* < 0.05
Jimenez 2012 [65]	USA, HPFS study, 20y FU (1986-NA)	Binary variable PD: self-reported; Tooth loss: self-reported	T2DM: self-reported T2DM (*N* = 2285) Control: non-T2DM (N = 32,962)	T2DM led to a 29% elevated risk for incident PD. Adjusted RR = 1.29, 95%CI 1.13–1.47 T2DM led to a 9% elevated risk for incident tooth loss. Adjusted RR = 1.09, 95%CI 1.01–1.18
Morita 2012 [68]	Japan, 5y FU (1997–2006)	Binary variable PD: CPI ≥ 3 Non-PD: CPI < 3	T2DM: HbA1c ≥ 6.5% (*N* = 150) Control: HbA1c < 6.5% (*N* = 5706)	T2DM led to a 17% elevated risk for incident PD. Adjusted RR = 1.17, 95%CI 1.01–1.36
Nelson 1990 [39]	USA, Pima Indians study, Mean 2.6y FU (1983–1989)	Binary variable PD: < 24 teeth present;> 6 teeth with ≥25% bone loss and any tooth with ≥50% bone loss. Non-PD: ≥24 teeth present; < 6 could have 25–50% bone loss and the rest < 25% bone loss	T2DM: OGTT ≥11.1 mM(*N* = 56) Control: no T2DM (*N* = 645)	T2DM led to a 160% elevated risk for incident PD. Adjusted RR = 2.57, 95%CI 1.0–6.6, p < 0.05
Taylor 1998 [69]	USA, Pima Indians study, Mean 2.3y FU (1.2–6.9 years)	Mean alveolar bone loss bone scores corresponded to bone loss of 0, 1to 24%, 25 to 49%, 50 to 74%, or > 75%	Diagnosed by OGTT (> 200 mg/dl) Better controlled T2DM: HbA1c ≤ 9% (N = 7) Poorer controlled T2DM: HbA1c > 9% (*N* = 14) Control: no T2DM (*N* = 338)	Better controlled T2DM led to a 120% elevated risk for alveolar bone loss progression, but was not statistically significant. Adjusted OR = 2.2, 95%CI 0.7–6.5, *p* = 0.175 Poorer controlled T2DM led to a 1040% elevated risk for alveolar bone loss progression. Adjusted OR = 11.4, 95%CI 2.5–53.3
**PD/non-PD**				
Demmer 2008 [63]	USA, NHEFS study 17y FU (1971–1992)	T2DM: Death certificate; self-reported T2DM and received anti-diabetes medications; facility discharge diagnosis	Category of baseline periodontal index, control group was the participants with lowest RPI score	Compared to the control group, participants in the 1st or 2nd categories did not experience an increased OR of developing T2DM, whereas the odds increased sharply in the 3rd category (OR 2.08; *P* < 0.0001). The ORs in 4th (1.71; *P* = 0.003) and 5th (1.50; *P* = 0.06) categories abated but remained elevated and were not statistically significantly different from the odds for those in the 3rd category.
			PD: clinical diagnosed(*N* = 1662) Gingivitis: clinical diagnosed (*N* = 2135) Control: periodontium health (*N* = 3372)	PD led to a 50% elevated risk for incident T2DM. Adjusted OR≈1.50, 95%CI NA, p < 0.05 Gingivitis led to a 40% elevated risk for incident T2DM. Adjusted OR≈1.40, 95%CI NA, p < 0.05
			Exposure: LOT 25–31 (N=NA) Control: LOT 0–8 (N=NA)	Loss more teeth at baseline led to a 70% elevated risk for incident T2DM. Adjusted OR≈1.70, 95%CI NA, p < 0.05
Ide 2010 [64]	Japan, 6.3y FU (2000–2007)	T2DM: FBG ≥ 125 mg/dl	Exposure1: CPI = 4 (*N* = 490) Exposure2: CPI = 3 (*N* = 2167) Control: CPI < 3 (*N* = 3191)	CPI = 4 led to a 28% elevated risk for incident T2DM, but was not statistically significant. Adjusted HR = 1.28, 95%CI 0.89–1.86 CPI = 3 did not led to an elevated risk for incident T2DM. Adjusted HR = 1.00, 95%CI 0.77–1.30
			Exposure1: LOT> 3 (*N* = 748) Exposure2: 1 < LOT< 3 (*N* = 2265) Control: LOT = 0 (*N* = 2835)	Loss more than 3 teeth did not lead to an elevated risk for incident T2DM Adjusted HR = 0.98 95%CI 0.69–1.39 Loss 1 or 2 teeth did not lead to an elevated risk for incident T2DM. Adjusted HR = 1.02 95%CI 0.79–1.32
Kebede 2017 [66]	Germany, SHIP study 11.1y FU (1997–2012)	T2DM: Self-reported physician diagnosed T2DM or treatment with antidiabetic medication	Exposure: mean PPD 2.70–7.25 mm (N=NA) Control: mean PPD 0.95–1.97 mm (N=NA)	Deeper PPD did not lead to an elevated risk for incident T2DM. Adjusted incidence RR = 1.271 95% 0.782–2.065
			Exposure: mean CAL 3.15–12.25 mm (N=NA) Control: mean CAL 0–1.15 mm (N=NA)	Higher CAL did not lead to an elevated risk for incident T2DM. Adjusted incidence RR = 0.819 95%CI 0.489–1.370
Miyawaki 2016 [67]	Japan, My health up Study, all male 5y FU (2004–2009)	T2DM: self-reported T2DM and received anti-diabetes medications, or based on clinical test (FBG ≥ 126 mg/dl or HbA1C ≥ 6.5%)	Exposure: self-reported tooth loosening (*N* = 262) Control: without tooth loosening (*N* = 2207)	Tooth loosening led to a 73% elevated risk for incident T2DM. Adjusted RR = 1.73, 95%CI 1.18–2.53
			Exposure: self-reported gingival bleeding (*N* = 795) Control: without gingival bleeding (*N* = 1674)	Gingival bleeding led to a 23% elevated risk for incident T2DM, but was not statistically significant. Adjusted RR = 1.23, 95%CI 0.90–1.70
Morita 2012 [68]	Japan, 5y FU (1997–2006)	T2DM: HbA1c ≥ 6.5%	Exposure1: CPI = 4 (*N* = 1634) Exposure2: CPI = 3 (*N* = 4114) Control: CPI = 0 (*N* = 1647)	CPI = 4 led to a 245% elevated risk for incident T2DM. Adjusted RR = 3.45, 95%CI 1.08–11.02, *p* = 0.037 CPI = 3 led to a 145% elevated risk for incident T2DM, but was not statistically significant. Adjusted RR = 2.47, 95%CI 0.78–7.79, *p* = 0.122
Myllymki 2018 [70]	Finland, Cohort 1935 Survey, 15-18y FU (1990–2008)	T2DM: WHO 1995 criteria	Exposure1: PPD = 4-5 mm (N = 98) Exposure2: PPD > 6 mm (*N* = 91) Control: No deep pockets (N = 88)	Both two exposures did not increase the T2DM incidence. 4-5 mm PPD: adjusted RR = 1.32, 95%CI 0.69–2.53, p > 0.05 > 6 mm PPD: adjusted RR = 1.56, 95%CI 0.84–2.92, p > 0.05
Winning 2016 [71]	UK, PRIME study 7.8y FU (2001–2010)	T2DM: FBG ≥ 126 mg/dl and WHO criteria	Exposure1: moderate PD Exposure2: severe PD Moderate/severe PD total = 553 Control: No significant PD (*N* = 778) PD severity was based on CDC/AAP classification	Moderate PD led to a 53% elevated risk for developing T2DM, but was not statistically significant. Adjusted RR = 1.53, 95%CI 0.86–2.74, p > 0.05 Severe PD led to an 85% elevated risk for developing T2DM Adjusted RR = 1.85, 95%CI 1.06–3.22, p < 0.05

PD: periodontitis; T2DM: type 2 diabetes mellitus; CAL: clinical attachment loss; PPD: periodontal pocket depth; LOT: loss of teeth; OGTT: oral glucose tolerance test; HbA1c: glycated hemoglobin; FBG: fasting plasma glucose; CI: confidence intervals; OR: odds ratio; RR: risk ratios; HR: hazard ratio; CPI: community periodontal index; RPI: Russell periodontal index

**Fig. 3:**
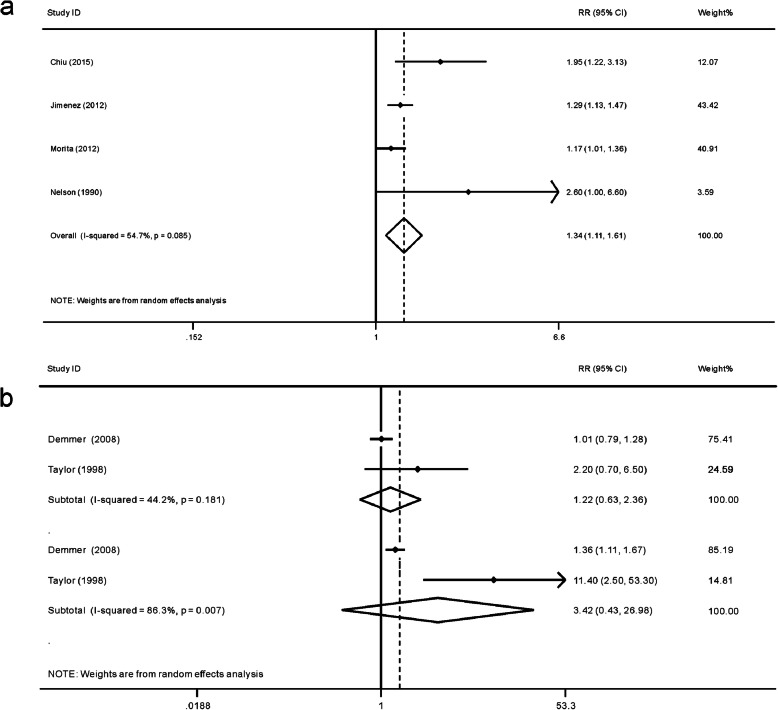
The impact of T2DM on periodontitis incidence. (**a**) Meta-analysis of periodontitis incidence (**b**) Meta-analysis of periodontitis incidence based on the level of glycemic control

Four studies investigating whether manifested T2DM increases periodontitis incidence were included in one meta-analysis. In total, 46,191 participants, including 2548 T2DM patients, were included, with a follow-up period ranging from 2.6 to 20 years. A total of 6361 incident periodontitis cases were detected. The results showed that T2DM led to a 34% elevated risk for incident periodontitis (RR = 1.34, 95% CI 1.11–1.61, *p* = 0.002, Fig. 3a). Slight heterogeneity among studies was detected (I^2^ = 54.7%, *p* = 0.085). Influence analysis found that this result was stable (Fig. S2a).

Another meta-analysis was carried out to investigate the impact of well-controlled and poorly controlled T2DM on periodontitis incidence. In total, two studies with 2791 participants were included. Ninety-four well-controlled and 89 poorly controlled T2DM patients at the baseline were selected as the exposure group. The follow-up was 2.3 (1.2–6.9) and 5 years, respectively. Two included studies [23, 69] both indicated that well-controlled T2DM did not increase the risk of periodontitis, and poorly controlled T2DM significantly promoted the incidence of periodontitis. The meta-analysis showed the same trend (Fig. 3b), but the results were non-significant for both well-controlled T2DM (RR = 1.22, 95% CI 0.63–2.39, *p* = 0.548) and poorly controlled T2DM (RR = 3.42, 95% CI 0.43–26.98, *p* = 0.243). The non-significant result of the latter might be caused by a high level of heterogeneity (*p* = 0.007; I^2^ = 86.3%).

#### Q3: does periodontitis increase the risk of developing T2DM?

A total of 7 cohort studies were included. The results are summarized in Table 2. In total, 27,498 participants were included. Among these participants, 8701 had mild periodontitis, while 3994 had severe periodontitis. A total of 1772 incident T2DM cases were detected during a follow-up period ranging from 5 to 18 years. Interestingly, all the included studies reported their results based on periodontitis severity. Thus, we performed two meta-analyses according to periodontitis severity as follows.

### The impact of mild periodontitis on T2DM incidence

**Fig. 4:**
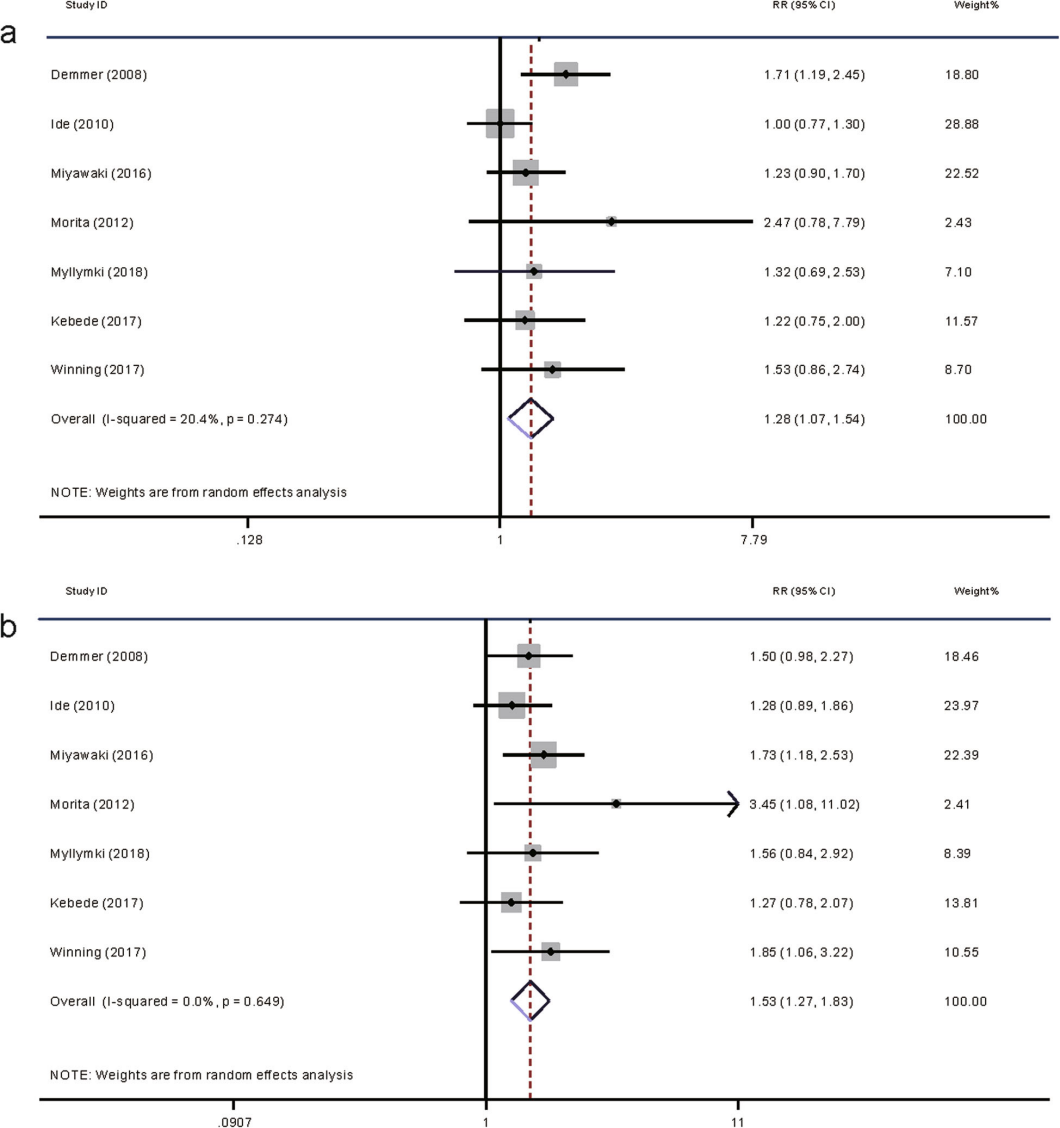
The impact of periodontitis on T2DM incidence. (**a**) Meta-analysis based on mild periodontitis (**b**) Meta-analysis based on severe periodontitis

A meta-analysis on this topic showed that mild periodontitis led to a 28% elevated risk for incident T2DM (RR = 1.28, 95% CI 1.07–1.54, *p* = 0.007, Fig. 4a). No significant heterogeneity (I^2^ = 20.4%, *p* = 0.27) or publication bias (Egger, *p* = 0.133; Begg, p = 0.133) among studies was detected. Influence analysis found that this result was unstable (Fig. S2b). Deleting Demmer’s study [63] would reduce the effect size and obtain a marginally significant result (RR = 1.17, 95% CI 0.99–1.39, *p* > 0.05). Due to this unstable result, we used the trim and fill method. After adding 3 hypothetical studies, the results became significant (RR = 1.14, 95% CI 0.92–1.41, *p* = 0.23, Table S5). The above results indicate that the effect of mild periodontitis on T2DM incidence was not very robust.

### The impact of severe periodontitis on T2DM incidence

Pooled results showed that severe periodontitis increased the risk of T2DM incidence by 53% (RR = 1.53, 95% CI 1.27–1.83, *p* = 0.000, Fig. 4b). The heterogeneity was very low (I^2^ = 0%, *p* = 0.649). No publication bias (Egger, *p* = 0.104; Begg, *p* = 0.230) was detected. In contrast to mild periodontitis, influence analysis found that the impact of severe periodontitis was very stable (Fig. S2c). To further confirm this, we used the trim and fill method. After adding 2 hypothetical studies, the results were still significant (RR = 1.46, 95% CI 1.23–1.73, p = 0.000, Table S5). The above results indicated that the effect of severe periodontitis on T2DM incidence was strong.

## Discussion

In this systematic review, we summarized observational studies exploring the bidirectional relationship between periodontitis and T2DM. Cross-sectional studies supported that there was a strong connection between periodontitis and T2DM. Prospective studies supported that T2DM and periodontitis promoted the incidence of each other and were related to disease severity. The strength of our work mainly lies in including the most up-to-date evidence and analyzing sufficient studies and participants. However, the limitations of our work are also worth noting.

For cross-sectional studies (Q1), high levels of heterogeneity existed among studies in 4 of our 6 meta-analyses. However, we did not find significant covariates that could decrease heterogeneity. Several reasons could partially explain the heterogeneity. First, these meta-analyses included a large number of studies, which would inevitably result in significant statistical diversity and cause statistical heterogeneity. Second, heterogeneity may result from measurement diversity. For example, the definitions of periodontitis were distinct, which could be based on a CPI code or clinical signs and symptoms. For CAL and PPD, measurement diversity was evident for the selection of teeth and probing sites. Third, the unreported confounding factors also caused heterogeneity. In contrast, in the 2 meta-analyses with limited heterogeneity based on adjusted ORs, the other 4 meta-analyses with high heterogeneity were all based on crude data. Few of the included studies reported confounding factors. This might partially explain why our meta-regression did not produce statistically significant results.

For cohort studies, we summarized that T2DM and periodontitis promoted the incidence of each other. This bidirectional promotion phenomenon was closely related to the severity of the ailment. We found that T2DM patients with a poorly controlled glycemic state tended to have a higher risk of suffering from periodontitis compared to patients with better glycemic control. For patients with severe periodontitis, the incidence of T2DM was significantly higher compared to those with mild periodontitis. However, this conclusion was drawn from the subgroup analysis of a limited number of studies. To further confirm this, generalized least-squares trend estimation [72, 73] or meta-regression should be used to evaluate this relationship. However, due to the inconsistency of exposure/outcome selection among limited studies, these analyses could not be performed. It is also worth noting that the same phenomenon was also revealed in the adjusted results of cross-sectional studies (Table 1) to a certain degree.

Several important works, though notable, were not included in our study. Chiu’s study [62] and Joshipura’s study [74] found that periodontitis could increase the risk of developing prediabetes. Demmer’s study [75] found that periodontitis was associated with 5-year HbA1c progression. Additionally, in the present work, we did not include studies focusing on other aspects of the connection between periodontitis and T2DM. Very recently, the joint workshop between the European Federation of Periodontology and the International Diabetes Federation updated a systematic review on the effect of periodontitis on diabetes [76]. In contrast to our present study, which focused on whether periodontitis and T2DM were significantly correlated, this systematic review mainly focused on how periodontitis influences T2DM progression. The authors concluded that for T2DM patients, periodontitis is associated with higher levels of HbA1c and significantly worse diabetes-related complications. This article counters the limitations of our work to some degree, and the details are undeniably valuable.

For future studies, several study design considerations should be considered. In our included studies, some researchers [21, 30, 34, 37] defined their studies as case-control studies by mistake. The control group was age- and sex-matched with the cases; however, the cases (T2DM patients) were not newly diagnosed but were diagnosed years earlier. Both T2DM and periodontitis are chronic diseases that cannot be cured, and they might aggravate each other via positive feedback. Thus, once selected participants have suffered from T2DM for years, this relationship could become perplexing since their worsened periodontal health could be regarded as the cause of T2DM as well as the effect of T2DM. Therefore, the design of these studies should not be regarded as case-control; actually, they should be considered to have a case-matched cross-sectional design since one could not distinguish the onset time of T2DM or periodontitis. This is also relevant for cohort studies. Incident outcomes, especially T2DM, reported within 1 year of the baseline should be excluded to minimize the prevalence of undiagnosed baseline T2DM [63, 71]. This also indicates that a longer follow-up period of cohort studies investigating these two diseases is required.

As demonstrated by the included studies with adjusted results, the significant confounding factors in this bidirectional relationship included age, sex, body mass index, waist circumference, C-reactive protein, white blood cell count, hypertension, triglyceride, smoking status, education, income, frequency of dentist visits and other data. To deepen the knowledge of this bidirectional relationship between periodontitis and T2DM, we suggest that future observational studies should take these confounding factors into consideration. For researchers, these confounders should be recorded, described and analyzed in detail. In addition, there was a trend that this bidirectional relationship might be related to disease severity. Future studies could investigate these details and use subgroup or regression analysis.

Based on the current available evidence, we concluded that periodontitis and T2DM had strong connections. Our findings suggest that dentists should be aware that periodontitis might indicate undiagnosed T2DM and poor glycemic control in T2DM patients; physicians should know the clinical signs of periodontitis to help T2DM patients improve their oral hygiene care and consider recommending periodontal therapy to improve glycemic control; patients should be aware that periodontitis and T2DM are risk factors for each other. Routine oral hygiene care and physical examinations are necessary for early prevention of T2DM or periodontitis.

## Conclusions

Based on the available evidence, we find an evident bidirectional relationship between T2DM and periodontitis. However, the number of cohort studies is limited. Further well-designed cohort studies, especially those investigating the impact of glycemic control state of T2DM on the incidence of periodontitis, are needed to confirm this finding. Our results suggest that both dentists and physicians need to be aware of the strong connection between periodontitis and T2DM. Also, it is reasonable that controlling these two diseases might help prevent each other’s incidence. DM: Diabetes mellitus.

## Supplementary information

**Additional file 1.** Appendix Table S1 Characteristics of the included cross-sectional studies. Appendix Table S2 Characteristics of the included cohort studies. Appendix Table S3 AHRQ scores of the cross-sectional studies. Appendix Table S4 NOS scores of the included cohort studies. Appendix Table S5 Summary of the trim and fill method

**Additional file 2.** Appendix Fig. S1 Influence analyses of cross-sectional studies (a) Results of adjusted ORs on T2DM prevalence (b) Results of adjusted ORs on periodontitis prevalence (c) Results of crude CAL (d) Results of crude PPD (e) Results of crude NOT (f) Results of crude LOT

**Additional file 3.** Appendix Fig. S2 Influence analyses of cohort studies (a) The impact of T2DM on periodontitis incidence (b) The impact of mild periodontitis on T2DM incidence (c) The impact of severe periodontitis on T2DM incidence

## Data Availability

All data generated or analyzed during the present study are included in this published article.

## Abbreviations

T2DM: Type 2 diabetes mellitus
RCTs: Randomized controlled trials
HbA1c: Glycated hemoglobin
CAL: Clinical attachment loss
PPD: Periodontal pocket depth
NOT: Number of teeth
LOT: Loss of teeth
CPI: Community periodontal index
OGTT: Oral glucose tolerance test
FBG: Fasting plasma glucose
NOS: Newcastle-Ottawa Scale
AHRQ: Agency for Healthcare Research and Quality
WMDs: Weighted mean differences
CIs: Confidence intervals
ORs: Odds ratios
RRs: Risk ratios
