# Supplementary material for: Epidemiologic relationship between periodontitis and type 2 diabetes mellitus
Source: BMC Oral Health. 2020 Jul 11;20:204. doi: 10.1186/s12903-020-01180-w (PMC7353775; doi:10.1186/s12903-020-01180-w)
Supplement: Supplementary file 1 — Additional file 1. Appendix Table S1 Characteristics of the included cross-sectional studies. Appendix Table S2 Characteristics of the included cohort studies. Appendix Table S3 AHRQ scores of the cross-sectional studies. Appendix Table S4 NOS scores of the included cohort studies. Appendix Table S5 Summary of the trim and fill method [file 12903_2020_1180_MOESM1_ESM.docx]

**Table S1 Characteristics of included cross-sectional studies**

| Investigator | Year | country | Number (case/control) | Age case | Age control | Male% case | Male% control | Recruitment (case) | Recruitment (control) | Mean HbA1c(mM) | T2DM period | Recruitment period | AHRQ  score | Outcome |
| --- | --- | --- | --- | --- | --- | --- | --- | --- | --- | --- | --- | --- | --- | --- |
| **PD/non-PD** | | | | | | | | | | | | | | |
| Awuti[1] | 2012 | China | 453/509 | NA | NA | 58.4 | 41.6 | P | P | NA | NA | NA | 7 | 1,6 |
| Choi[2] | 2011 | America | 12254 | NA | NA | NA | NA | NHANES | NHANES | NA | NA | NA | 8 | 0 |
| Nesse[3] | 2010 | Netherland | 217/320 | 41 | 33 | 52 | 44 | D | D | NA | NA | NA | 7 | 1,6 |
| Saito[4] | 2005 | Japan | 584 | NA | NA | NA | NA | P | P | NA | NA | 6m | 6 | 0 |
| **T2DM/non-T2DM** | | | | | | | | | | | | | | |
| Abduljabbar[5] | 2017 | Saudi Arabia | 43/87 | 50.6 | 52.3 | NA | NA | P | P | 8.4 | 3.1y | NA | 6 | 3,5 |
| Campus[6] | 2005 | Italy | 71/141 | 61 | 59.1 | 44 | 43 | E | D | 7.5 | >8y | NA | 6 | 1 |
| Demmer[7] | 2012 | Germany | 152/2280 | 59.6 | 44 | 46 | 64 | SHIP | SHIP | 7.18 | NA | 1y | 8 | 2,3,4 |
| Hintao[8] | 2007 | Thailand | 105/103 | 54.3 | 53.3 | 49.5 | 49.5 | E | H | NA | NA | NA | 7 | 2 |
| Kaur[9] | 2009 | Germany | 182/1314 | 64.5 | 61 | 57.1 | 50.4 | SHIP | SHIP | NA | 10y | NA | 8 | 2,3,5 |
| Khader[10] | 2008 | Jordan | 106/106 | 46.9 | 47 | 46.3 | 50.9 | H | H | NA | NA | 4m | 6 | 2,3,5 |
| Kowall[11] | 2015 | Germany | 777/2145 | 56.9 | 44.8 | 56.4 | 46.3 | SHIP | SHIP | 6.66 | NA | 4y | 8 | 0 |
| Lan[12] | 2012 | China | 105/105 | 41.5 | 42.1 | 57.1 | 52.4 | D | D | NA | 1-7y | 1y | 4 | 2,3 |
| Leong[13] | 2007 | USA | 51/102 | 65.06 | 64.01 | 37.25 | 37.25 | H | H | 7.39 | NA | 2y | 6 | 5 |
| Leung[14] | 2008 | China | 161/364 | 64.1 | 63.6 | 46.6 | 46.2 | E | H | 7.9 | 2.5y | 1.5m | 7 | 1,5,6 |
| Liao[15] | 2010 | China | 118/50 | 62 | 62 | 64.4 | 60 | H | H | NA | NA | 4m | 5 | 2,3,5 |
| Liu[16] | 2013 | China | 106/440 | >65 | >65 | 47.2 | 45 | H | H | NA | >6y | 1m | 7 | 1,2 |
| Lopez-Lopez[17] | 2011 | Spain | 50/50 | 60.7 | 61.6 | 40 | 44 | D | D | 6.6 | NA | NA | 4 | 4 |
| Mansour[18] | 2005 | Iraq | 633/960 | 56.7 | 56.9 | 46.6 | 43.8 | E | H | NA | NA | 1y6m | 6 | 1 |
| Marotta[19] | 2012 | Brazil | 30/60 | 58.2 | 58.3 | 40 | NA | D | D | NA | NA | NA | 4 | 4 |
| Mattout[20] | 2006 | France | 71/2061 | 54.5 | 49 | 59.1 | 48.7 | P | P | 8.45 | NA | 1y | 7 | 2,3 |
| Mohamed[21] | 2013 | Norway | 154/303 | 52.6 | 52.4 | 39 | 39.3 | D | D | NA | 9y,>1y | 6m | 8 | 1,6 |
| Morton[22] | 1995 | UK | 24/24 | 46.8 | 45.8 | 33.33 | 62.5 | D | D | NA | 7.9y, 2y | NA | 4 | 2,3 |
| Nelson[23] | 1990 | America | 720/1553 | NA | NA | NA | NA | P | P | NA | NA | 2y | 8 | 1,6 |
| Ou[24] | 2013 | China | 54/54 | 60 | 65 | 51.85 | 62.96 | E | H | 7.3 | 8y, >1y | 2y | 5 | 2,5 |
| Patino[25] | 2008 | Mexico | 35/35 | 45 | 42 | 40 | 39 | D | D | 8 | >5y | 3y | 5 | 2,3,5 |
| Preshaw[26] | 2010 | Sri Lankan | 285/60 | 45.7 | 45.1 | 51.2 | 43.3 | E | P | NA | NA | NA | 5 | 1,2,3,5 |
| Qin[27] | 2014 | China | 52/48 | 64.27 | 65.08 | 63.46 | 58.33 | E | H | NA | NA | 1y | 4 | 1,2,3 |
| Saito[28] | 2004 | Japan | 101/850 | 60.5 | 56.8 | 60.4 | 37.2 | P | P | 6.4 | NA | 4m | 6 | 2,3,4 |
| Saito[29] | 2006 | Japan | 131/62 | 52.1 | 52.3 | 100 | 100 | H | H | NA | NA | 8m | 6 | 4 |
| Serrano[30] | 2012 | Colombia | 72/39 | 61.59 | 59.17 | 50 | 33.33 | P | P | NA | NA | NA | 6 | 2,3,4 |
| Sun[31] | 2009 | China | 657/649 | 68.5 | 67.9 | 45.81 | 45.92 | D | D | NA | >5 | 4y | 4 | 1 |
| Susanto[32] | 2011 | Netherland | 78/65 | 56.7 | 50.5 | 45 | 34 | D/E | D/E | NA | NA | 8m | 7 | 2,3,4 |
| Tanwir[33] | 2009 | Pakistan | 88/80 | 41.8 | 41.3 | 50 | 43 | P | P | NA | NA | NA | 6 | 1,4 |
| Tsai[34] | 2002 | USA | 303/4040 | NA | NA | 70.6 | 48.9 | NHANES III | NHANES III | NA | NA | NA | 4 | 6 |
| Wang[35] | 2009 | Taiwan | 193/8468 | 40.01 | 39.33 | 43.5 | 32.09 | KCIS | KCIS | NA | 4.12y | 4y | 8 | 1,6 |
| Wang[36] | 2015 | China | 50/50 | 72.5 | 73.5 | NA | NA | P | P | NA | NA | 1y | 4 | 1 |
| Yuan[37] | 2001 | Taiwan | 105/141 | 57 | 56.95 | 57.14 | 58.16 | P | P | NA | NA | NA | 7 | 2,3 |
| Zhang[38] | 2012 | China | 44/43 | 51.1 | 51.16 | 52.27 | 51.16 | E | H | NA | >3y | 5m | 5 | 3 |
| Zhang[39] | 2013 | China | 60/90 | 86.35 | 86.93 | 100 | 100 | P | P | 7.27 | >5y | 2m | 4 | 1 |
| Zheng[40] | 2016 | China | 54/50 | 65.8 | 66.6 | 51.85 | 52 | E | D | NA | >1y | 1y | 4 | 2,3 |
| Zhou[41] | 2013 | China | 63/63 | 60.24 | 61.68 | 44.44 | 42.86 | E | H | NA | 8y,1-30y | 1y2m | 5 | 5 |
| Zielinski[42] | 2002 | America | 32/40 | 71 | 74 | 37 | 45 | D | H | 7.3 | 5-15y | NA | 4 | 5 |
| Zou[43] | 2014 | China | 94/420 | 52.36 | 50.82 | 54.25 | 63.57 | D | D | NA | 5y | 2y5m | 4 | 1 |

NA: not available; E: endocrine department; D: dental clinic; P: population; H: hospital; PD: periodontitis; T2DM: type 2 diabetes mellitus

0: adjusted data not included into meta-analyses and summarized in table 1. 1-5: unadjusted data of OR, CAL, PPD, NOT, LOT, included into meta-analyses. 6: adjusted data included into meta-analyses.

**Table S2 Characteristics of included cohort studies**

| Investigator | Year | Country | Number | Incident Cases | Follow Up | Age | Male% | Longitudinal Study | NOS Score |
| --- | --- | --- | --- | --- | --- | --- | --- | --- | --- |
| **T2DM/non-T2DM** | | | | | | | | | |
| Chiu[44] | 2015 | Taiwan | 4387 | 1247 | 5 | 35-44 | 30.80% | KCIS | 7 |
| Demmer[7] | 2012 | Germany | 2626 | NA | 5 | 46 | 48% | SHIP | 8 |
| Jimenez[45] | 2012 | USA | 35247 | 3009 | 20 | 54 | All Male | HPFS | 6 |
| Morita[46] | 2012 | Japan | 5856 | 2068 | 5 | 30-69 | 77% | NA | 8 |
| Nelson[23] | 1990 | USA | 701 | 37 | 2.6 | 15-54 | ≈40% | Pima Indians study | 6 |
| Taylor[47] | 1998 | USA | 359 | 151 | 2.3 (1.2-6.9) | 15-57 | 40% | Pima Indians study | 5 |
| **PD/non-PD** | | | | | | | | | |
| Demmer[48] | 2008 | USA | 9296 | 817 | 17±4 | 55 | 40% | NHANES I NHEFS | 7 |
| Ide[49] | 2010 | Japan | 5848 | 287 | 6.5 (2-7) | 43 | 66.40% | NA | 8 |
| Kebede[50] | 2017 | Germany | 2034 | 206 | 11.1 | 45.4 | 46% | SHIP | 6 |
| Miyawaki[51] | 2016 | Japan | 2469 | 133 | 5 | 45 | All Male | My health up | 5 |
| Morita[46] | 2012 | Japan | 6125 | 168 | 5 | 30-69 | 82.40% | NA | 8 |
| Myllymki[52] | 2018 | Finland | 395 | 81 | 15-18 | 55-57 | 46% | Cohort 1935 Survey | 4 |
| Winning[53] | 2016 | UK | 1331 | 80 | 7.8 | 63.7 | All Male | PRIME study | 5 |

NA: not available; PD: periodontitis; T2DM: type 2 diabetes mellitus

**Table S3 AHRQ scores of cross-sectional studies**

| Study | Year | Ref | 1 | 2 | 3 | 4 | 5 | 6 | 7 | 8 | 9 | 10 | 11 | Total |
| --- | --- | --- | --- | --- | --- | --- | --- | --- | --- | --- | --- | --- | --- | --- |
| **PD/non-PD** | | | | | | | | | | | | | | |
| Awuti | 2012 | 1 | 1 | 1 | 1 | 0 | 1 | 1 | 0 | 1 | 1 | 0 | 0 | 7 |
| Choi | 2011 | 2 | 1 | 1 | 0 | 1 | 1 | 1 | 1 | 1 | 1 | 0 | 0 | 8 |
| Nesse | 2010 | 3 | 1 | 1 | 0 | 1 | 1 | 1 | 1 | 0 | 1 | 0 | 0 | 7 |
| Saito | 2005 | 4 | 1 | 0 | 1 | 1 | 0 | 1 | 1 | 1 | 0 | 0 | 0 | 6 |
| Abduljabbar | 2017 | 5 | 1 | 1 | 1 | 0 | 1 | 1 | 0 | 1 | 0 | 0 | 0 | 6 |
| Campus | 2005 | 6 | 1 | 1 | 0 | 0 | 1 | 1 | 1 | 1 | 0 | 0 | 0 | 6 |
| **T2DM/non-T2DM** | | | | | | | | | | | | | | |
| Demmer | 2012 | 7 | 1 | 1 | 0 | 1 | 1 | 0 | 1 | 1 | 1 | 1 | 0 | 8 |
| Hintao | 2007 | 8 | 1 | 1 | 1 | 0 | 1 | 1 | 1 | 1 | 0 | 0 | 0 | 7 |
| Kaur | 2009 | 9 | 1 | 1 | 0 | 1 | 1 | 0 | 1 | 1 | 1 | 1 | 0 | 8 |
| Khader | 2008 | 10 | 1 | 1 | 1 | 1 | 1 | 0 | 0 | 1 | 0 | 0 | 0 | 6 |
| Kowall | 2015 | 11 | 1 | 1 | 1 | 1 | 1 | 0 | 1 | 1 | 0 | 1 | 0 | 8 |
| Lan | 2012 | 12 | 1 | 1 | 1 | 1 | 0 | 0 | 0 | 0 | 0 | 0 | 0 | 4 |
| Leong | 2007 | 13 | 1 | 1 | 1 | 1 | 0 | 0 | 1 | 1 | 0 | 0 | 0 | 6 |
| Leung | 2008 | 14 | 1 | 1 | 1 | 1 | 1 | 0 | 1 | 1 | 0 | 0 | 0 | 7 |
| Liao | 2010 | 15 | 1 | 1 | 1 | 1 | 0 | 0 | 0 | 1 | 0 | 0 | 0 | 5 |
| Liu | 2013 | 16 | 1 | 1 | 1 | 1 | 1 | 0 | 0 | 1 | 0 | 1 | 0 | 7 |
| Lopez-Lopez | 2011 | 17 | 1 | 1 | 0 | 0 | 1 | 1 | 0 | 0 | 0 | 0 | 0 | 4 |
| Mansour | 2005 | 18 | 1 | 0 | 1 | 1 | 1 | 0 | 1 | 0 | 1 | 0 | 0 | 6 |
| Marotta | 2012 | 19 | 1 | 0 | 1 | 0 | 1 | 1 | 0 | 0 | 0 | 0 | 0 | 4 |
| Mattout | 2006 | 20 | 1 | 1 | 1 | 1 | 0 | 1 | 1 | 0 | 1 | 0 | 0 | 7 |
| Mohamed | 2013 | 21 | 1 | 1 | 1 | 1 | 1 | 1 | 1 | 1 | 0 | 0 | 0 | 8 |
| Morton | 1995 | 22 | 1 | 1 | 0 | 0 | 1 | 1 | 0 | 0 | 0 | 0 | 0 | 4 |
| Nelson | 1990 | 23 | 1 | 1 | 1 | 1 | 1 | 0 | 1 | 0 | 1 | 1 | 0 | 8 |
| Ou | 2013 | 24 | 1 | 1 | 1 | 1 | 1 | 0 | 0 | 0 | 0 | 0 | 0 | 5 |
| Patino | 2008 | 25 | 1 | 1 | 1 | 1 | 0 | 1 | 0 | 0 | 0 | 0 | 0 | 5 |
| Preshaw | 2010 | 26 | 1 | 1 | 0 | 0 | 1 | 1 | 0 | 1 | 0 | 0 | 0 | 5 |
| Qin | 2014 | 27 | 1 | 1 | 1 | 1 | 0 | 0 | 0 | 0 | 0 | 0 | 0 | 4 |
| Saito | 2004 | 28 | 1 | 1 | 1 | 0 | 1 | 1 | 0 | 1 | 0 | 0 | 0 | 6 |
| Saito | 2006 | 29 | 1 | 1 | 1 | 0 | 1 | 1 | 0 | 1 | 0 | 0 | 0 | 6 |
| Serrano | 2012 | 30 | 1 | 1 | 0 | 1 | 1 | 0 | 1 | 1 | 0 | 0 | 0 | 6 |
| Sun | 2009 | 31 | 1 | 1 | 1 | 1 | 0 | 0 | 0 | 0 | 0 | 0 | 0 | 4 |
| Susanto | 2011 | 32 | 1 | 1 | 1 | 1 | 1 | 0 | 1 | 1 | 0 | 0 | 0 | 7 |
| Tsai | 2002 | 33 | 1 | 1 | 1 | 0 | 0 | 0 | 1 | 0 | 0 | 0 | 0 | 4 |
| Tanwir | 2009 | 34 | 1 | 1 | 0 | 1 | 1 | 0 | 0 | 1 | 0 | 1 | 0 | 6 |
| Wang | 2009 | 35 | 1 | 1 | 1 | 1 | 1 | 1 | 0 | 1 | 1 | 0 | 0 | 8 |
| Wang | 2015 | 36 | 1 | 1 | 1 | 1 | 0 | 0 | 0 | 0 | 0 | 0 | 0 | 4 |
| Yuan | 2001 | 37 | 1 | 1 | 1 | 1 | 1 | 1 | 0 | 0 | 1 | 0 | 0 | 7 |
| Zhang | 2012 | 38 | 1 | 1 | 1 | 1 | 0 | 1 | 0 | 0 | 0 | 0 | 0 | 5 |
| Zhang | 2013 | 39 | 1 | 1 | 1 | 1 | 0 | 0 | 0 | 0 | 0 | 0 | 0 | 4 |
| Zheng | 2016 | 40 | 1 | 1 | 1 | 1 | 0 | 0 | 0 | 0 | 0 | 0 | 0 | 4 |
| Zhou | 2013 | 41 | 1 | 1 | 1 | 1 | 1 | 0 | 0 | 0 | 0 | 0 | 0 | 5 |
| Zielinski | 2002 | 42 | 1 | 1 | 0 | 0 | 0 | 1 | 0 | 1 | 0 | 0 | 0 | 4 |
| Zou | 2014 | 43 | 1 | 1 | 1 | 1 | 0 | 0 | 0 | 0 | 0 | 0 | 0 | 4 |

Note: PD: periodontitis; T2DM: type 2 diabetes mellitus

AHRQ scoring category: 1. Define the source of information (survey, record review); 2. List inclusion and exclusion criteria for exposed and unexposed subjects (cases and controls) or refer to previous publications; 3. Indicate time period used for identifying patients; 4. Indicate whether or not subjects were consecutive if not population-based; 5. Indicate if evaluators of subjective components of study were masked to other aspects of the status of the participants; 6. Describe any assessments undertaken for quality assurance purposes (e.g., test/retest of primary outcome measurements); 7. Explain any patient exclusions from analysis; 8. Describe how confounding was assessed and/or controlled; 9. If applicable, explain how missing data were handled in the analysis; 10. Summarize patient response rates and completeness of data collection; 11. Clarify what follow-up, if any, was expected and the percentage of patients for which incomplete data or follow-up was obtained.

**Table S4 NOS scores of cohort studies**

| **T2DM/non-T2DM** | | | | | | | | | | | |
| --- | --- | --- | --- | --- | --- | --- | --- | --- | --- | --- | --- |
| Study | Year | Ref | 1 | 2 | 3 | 4 | 5 | 6 | 7 | 8 | Total |
| Chiu | 2015 | 44 | 1 | 1 | 1 | 1 | 2 | 0 | 1 | 0 | 7 |
| Demmer | 2012 | 7 | 1 | 1 | 1 | 1 | 2 | 0 | 1 | 1 | 8 |
| Jimenez | 2012 | 45 | 0 | 1 | 1 | 1 | 2 | 0 | 1 | 0 | 6 |
| Morita | 2012 | 46 | 1 | 1 | 1 | 1 | 2 | 1 | 1 | 0 | 8 |
| Nelson | 1990 | 23 | 0 | 1 | 1 | 1 | 1 | 1 | 0 | 1 | 6 |
| Taylor | 1998 | 47 | 0 | 1 | 1 | 1 | 1 | 0 | 0 | 1 | 5 |
| **PD/non-PD** | | | | | | | | | | | |
| Demmer | 2008 | 48 | 1 | 1 | 1 | 1 | 2 | 0 | 1 | 0 | 7 |
| Ide | 2010 | 49 | 1 | 1 | 0 | 1 | 2 | 1 | 1 | 1 | 8 |
| Kebede | 2017 | 50 | 1 | 1 | 0 | 1 | 2 | 0 | 1 | 0 | 6 |
| Miyawaki | 2016 | 51 | 0 | 1 | 0 | 1 | 2 | 0 | 1 | 0 | 5 |
| Morita | 2012 | 46 | 1 | 1 | 1 | 1 | 2 | 1 | 1 | 0 | 8 |
| Myllymki | 2018 | 52 | 1 | 1 | 0 | 1 | 1 | 0 | 0 | 0 | 4 |
| Winning | 2016 | 53 | 0 | 1 | 1 | 1 | 2 | 0 | 0 | 0 | 5 |

Note: PD: periodontitis; T2DM: type 2 diabetes mellitus

NOS scoring category: 1.Representativeness of the exposed cohort; 2. Selection of the non-exposed cohort; 3. Ascertainment of exposure; 4. Demonstration that outcome of interest was not present at start of study; 5. Comparability of cohorts on the basis of the design or analysis; 6. Assessment of outcome; 7. Was follow-up long enough for outcomes to occur; 8. Adequacy of follow up of cohorts.

**Table S5 Summary of trim and fill method**

| Meta-analyses | Trim and filled method | | |
| --- | --- | --- | --- |
|  | Mixed model | Flip model | Default model |
| CAL differences | Add 6 studies (p=0.000 WMD=0.45) | Add 1 studies (p=0.000 WMD=0.95) | Add 0 study |
| PPD differences | Add 4 studies (p=0.000 WMD=0.39) | Add 1 study (p=0.000 WMD=0.65) | Add 0 study |
| Mild PD on T2DM incidence | Add 0 study | Add 3 studies (p=0.23 RR=1.14) | Add 3 studies (p=0.23 RR=1.14) |
| Severe PD on T2DM incidence | Add 0 study | Add 2 studies (p=0.000 RR=1.46) | Add 2 studies (p=0.000 RR=1.46) |

**Reference list**

1. Awuti G, Younusi K, Li L, Upur H, Ren J (2012) Epidemiological survey on the prevalence of periodontitis and diabetes mellitus in Uyghur adults from rural Hotan area in Xinjiang. Exp Diabetes Res 2012:758921. doi:10.1155/2012/758921

2. Choi YH, McKeown RE, Mayer-Davis EJ, Liese AD, Song KB, Merchant AT (2011) Association between periodontitis and impaired fasting glucose and diabetes. Diabetes Care 34 (2):381-386. doi:10.2337/dc10-1354

3. Nesse W, Dijkstra PU, Abbas F, Spijkervet FK, Stijger A, Tromp JA, van Dijk JL, Vissink A (2010) Increased prevalence of cardiovascular and autoimmune diseases in periodontitis patients: a cross-sectional study. J Periodontol 81 (11):1622-1628. doi:10.1902/jop.2010.100058

4. Saito T, Shimazaki Y, Kiyohara Y, Kato I, Kubo M, Iida M, Yamashita Y (2005) Relationship between obesity, glucose tolerance, and periodontal disease in Japanese women: the Hisayama study. J Periodontal Res 40 (4):346-353. doi:10.1111/j.1600-0765.2005.00813.x

5. Abduljabbar T, Al-Sahaly F, Al-Kathami M, Afzal S, Vohra F (2017) Comparison of periodontal and peri-implant inflammatory parameters among patients with prediabetes, type 2 diabetes mellitus and non-diabetic controls. Acta Odontol Scand 75 (5):319-324. doi:10.1080/00016357.2017.1303848

6. Campus G, Salem A, Uzzau S, Baldoni E, Tonolo G (2005) Diabetes and periodontal disease: a case-control study. J Periodontol 76 (3):418-425

7. Demmer RT, Holtfreter B, Desvarieux M, Jacobs DR, Jr., Kerner W, Nauck M, Volzke H, Kocher T (2012) The influence of type 1 and type 2 diabetes on periodontal disease progression: prospective results from the Study of Health in Pomerania (SHIP). Diabetes Care 35 (10):2036-2042. doi:10.2337/dc11-2453

8. Hintao J, Teanpaisan R, Chongsuvivatwong V, Dahlen G, Rattarasarn C (2007) Root surface and coronal caries in adults with type 2 diabetes mellitus. Community Dent Oral Epidemiol 35 (4):302-309. doi:10.1111/j.1600-0528.2007.00325.x

9. Kaur G, Holtfreter B, Rathmann W, Schwahn C, Wallaschofski H, Schipf S, Nauck M, Kocher T (2009) Association between type 1 and type 2 diabetes with periodontal disease and tooth loss. Journal of clinical periodontology 36 (9):765-774. doi:10.1111/j.1600-051X.2009.01445.x

10. Khader YS, Albashaireh ZSM, Hammad MM Periodontal status of type 2 diabetics compared with nondiabetics in north Jordan. East Mediterr Health J. 2008 May-Jun;14(3):654-61.,

11. Kowall B, Holtfreter B, Volzke H, Schipf S, Mundt T, Rathmann W, Kocher T (2015) Pre-diabetes and well-controlled diabetes are not associated with periodontal disease: the SHIP Trend Study. Journal of clinical periodontology 42 (5):422-430. doi:10.1111/jcpe.12391

12. Lan B, Liu Y (2012) The characteristics of periodontal disease in diabetic patients and its control measures. J Med Theor & Prac 25 (21):2667-2668[Chinese]

13. Leong P, Tumanyan S, Blicher B, Yeung A, Joshipura K (2007) Periodontal Disease among Adult, New-Immigrant, Chinese Americans in Boston with and without Diabetes ? A Brief Communication. Journal of Public Health Dentistry 67 (3):171-173. doi:10.1111/j.1752-7325.2007.00042.x

14. Leung WK, Siu SC, Chu FC, Wong KW, Jin L, Sham AS, Tsang CS, Samaranayake LP (2008) Oral health status of low-income, middle-aged to elderly Hong Kong Chinese with type 2 diabetes mellitus. Oral Health Prev Dent 6 (2):105-118

15. Liao Y, He L, Li P (2010) Periodontal condition on patients with type 2 diabetes mellitus in urban area of Beijing Chin J Diabetes Mellitus 2 (4):248-252[Chinese]

16. Liu W, Li G, Yan N, Hu Y (2013) Oral health survey of the elderly pafients with diabetes in Sim jiamiao community in Xi'an. Chin J Diabetes Mellitus 11 (4):216-220

17. Lopez-Lopez J, Jane-Salas E, Estrugo-Devesa A, Velasco-Ortega E, Martin-Gonzalez J, Segura-Egea JJ (2011) Periapical and endodontic status of type 2 diabetic patients in Catalonia, Spain: a cross-sectional study. J Endod 37 (5):598-601. doi:10.1016/j.joen.2011.01.002

18. Mansour AA, Abd-Al-Sada N (2005) Periodontal disease among diabetics in Iraq. Medgenmed [Computer File]: Medscape General Medicine 7 (3)

19. Marotta PS, Fontes TV, Armada L, Lima KC, Rocas IN, Siqueira JF, Jr. (2012) Type 2 diabetes mellitus and the prevalence of apical periodontitis and endodontic treatment in an adult Brazilian population. J Endod 38 (3):297-300. doi:10.1016/j.joen.2011.11.001

20. Mattout C, Bourgeois D, Bouchard P (2006) Type 2 diabetes and periodontal indicators: epidemiology in France 2002-2003. J Periodontal Res 41 (4):253-258. doi:10.1111/j.1600-0765.2006.00862.x

21. Mohamed HG, Idris SB, Ahmed MF, Boe OE, Mustafa K, Ibrahim SO, Astrom AN (2013) Association between oral health status and type 2 diabetes mellitus among Sudanese adults: a matched case-control study. PLoS One 8 (12):e82158. doi:10.1371/journal.pone.0082158

22. Morton AA, Williams RW, Watts TL (1995) Initial study of periodontal status in non-insulin-dependent diabetics in Mauritius. J Dent 23 (6):343-345

23. Nelson RG, Shlossman M, Budding LM, Pettitt DJ, Saad MF, Genco RJ, Knowler WC (1990) Periodontal disease and NIDDM in Pima Indians. Diabetes Care 13 (8):836-840

24. Ou X, Zhou Y, Chen Y (2013) Investigation on the Oral Health Status of Patients with Type Ⅱ diabetes mellitus. Guangxi Med J 35 (12):1601-1603[Chinese]

25. Patino Marin N, Loyola Rodriguez JP, Medina Solis CE, Pontigo Loyola AP, Reyes Macias JF, Ortega Rosado JC, Aradillas Garcia C (2008) Caries, periodontal disease and tooth loss in patients with diabetes mellitus types 1 and 2. Acta odontologica latinoamericana : AOL 21 (2):127-133

26. Preshaw PM, de Silva N, McCracken GI, Fernando DJ, Dalton CF, Steen ND, Heasman PA (2010) Compromised periodontal status in an urban Sri Lankan population with type 2 diabetes. Journal of clinical periodontology 37 (2):165-171

27. Qin J (2014) Comparison of Periodontal Health and Oral Hygiene Type 2 Diabetes Patients in Community. Chin Prac Med 9 (32):228-229[Chinese]

28. Saito T, Shimazaki Y, Kiyohara Y, Kato I, Kubo M, Iida M, Koga T (2004) The severity of periodontal disease is associated with the development of glucose intolerance in non-diabetics: the Hisayama study. J Dent Res 83 (6):485-490

29. Saito T, Murakami M, Shimazaki Y, Matsumoto S, Yamashita Y (2006) The extent of alveolar bone loss is associated with impaired glucose tolerance in Japanese men. J Periodontol 77 (3):392-397. doi:10.1902/jop.2006.050061

30. Serrano C, Perez C, Rodriguez M (2012) Periodontal conditions in a group of Colombian type 2 diabetic patients with different degrees of metabolic control. Acta odontologica latinoamericana : AOL 25 (1):132-139

31. Sun L (2009) A survey of periodontitis, dentition defect and edentulous status in aged patients with diabetes mellitus. Chin J Geriatr Dent 7 (1):17-19[Chinese]

32. Susanto H, Nesse W, Dijkstra PU, Agustina D, Vissink A, Abbas F (2011) Periodontitis prevalence and severity in Indonesians with type 2 diabetes. J Periodontol 82 (4):550-557. doi:10.1902/jop.2010.100285

33. Tanwir F, Altamash M, Gustafsson A (2009) Effect of diabetes on periodontal status of a population with poor oral health. Acta Odontol Scand 67 (3):129-133. doi:10.1080/00016350802208406

34. Tsai C, Hayes C, Taylor GW (2002) Glycemic control of type 2 diabetes and severe periodontal disease in the US adult population. Community Dent Oral Epidemiol 30 (3):182-192

35. Wang TT, Chen TH, Wang PE, Lai H, Lo MT, Chen PY, Chiu SY (2009) A population-based study on the association between type 2 diabetes and periodontal disease in 12,123 middle-aged Taiwanese (KCIS No. 21). Journal of clinical periodontology 36 (5):372-379. doi:10.1111/j.1600-051X.2009.01386.x

36. Wang X (2015) Analysis of periodontitis and dentition defect in elderly diabetic patients. Diabetes New World (Mar):106[Chinses]. doi:10.16658/j.cnki.1672-4062.2015.06.165

37. Yuan K, Chang CJ, Hsu PC, Sun HS, Tseng CC, Wang JR (2001) Detection of putative periodontal pathogens in non-insulin-dependent diabetes mellitus and non-diabetes mellitus by polymerase chain reaction. Journal of Periodontal Research 36 (1):18-24

38. Zhang H, Huang Y (2012) Analysis of the Relationship between Periodontal Tissue Disease and Type 2 Diabetes Mellitus. Prim Med 16 (16):2096-2097

39. Zhang Y, Li H, Ren J (2013) The investigation on the Situation of periodontitis and Defect Dentition of Elder Men Patients with Diabetes Mellitus. Chin J Geriatr Dent 11 (5):281-283[Chinese]

40. Zheng X (2016) A certain community of type 2 diabetes in patients with chronic periodontitis periodontal health survey. Cuide Chin Med 14 (16):11-12[Chinese]

41. Zhou Y, Peng L, Liu H (2013) Clinical study on tooth loss and restoring and their related factors in patients with diabetes mellitus. Chin J New Clinical Med 6 (11):1046-1049[Chinese]

42. Zielinski MB, Fedele D, Forman LJ, Pomerantz SC (2002) Oral health in the elderly with non-insulin-dependent diabetes mellitus. Special Care in Dentistry 22 (3):94-98

43. Zou G (2014) The correlation and analysis of chronic periodontitis and type 2 diabetes mellitus. J Med Theor & Prac 27 (23):3225-3226[Chinese]

44. Chiu SY, Lai H, Yen AM, Fann JC, Chen LS, Chen HH (2015) Temporal sequence of the bidirectional relationship between hyperglycemia and periodontal disease: a community-based study of 5,885 Taiwanese aged 35-44 years (KCIS No. 32). Acta Diabetol 52 (1):123-131. doi:10.1007/s00592-014-0612-0

45. Jimenez M, Hu FB, Marino M, Li Y, Joshipura KJ (2012) Type 2 diabetes mellitus and 20 year incidence of periodontitis and tooth loss. Diabetes Res Clin Pract 98 (3):494-500. doi:10.1016/j.diabres.2012.09.039

46. Morita I, Inagaki K, Nakamura F, Noguchi T, Matsubara T, Yoshii S, Nakagaki H, Mizuno K, Sheiham A, Sabbah W (2012) Relationship between periodontal status and levels of glycated hemoglobin. J Dent Res 91 (2):161-166. doi:10.1177/0022034511431583

47. Taylor GW, Burt BA, Becker MP, Genco RJ, Shlossman M (1998) Glycemic control and alveolar bone loss progression in type 2 diabetes. Annals of Periodontology 3 (1):30-39

48. Demmer RT, Jacobs DR, Jr., Desvarieux M (2008) Periodontal disease and incident type 2 diabetes: results from the First National Health and Nutrition Examination Survey and its epidemiologic follow-up study. Diabetes Care 31 (7):1373-1379. doi:10.2337/dc08-0026

49. Ide R, Hoshuyama T, Wilson D, Takahashi K, Higashi T (2011) Periodontal disease and incident diabetes: a seven-year study. J Dent Res 90 (1):41-46. doi:10.1177/0022034510381902

50. Kebede TG, Pink C, Rathmann W, Kowall B, Volzke H, Petersmann A, Meisel P, Dietrich T, Kocher T, Holtfreter B (2017) Does periodontitis affect diabetes incidence and haemoglobin A1c change? An 11-year follow-up study. Diabetes & metabolism. doi:10.1016/j.diabet.2017.11.003

51. Miyawaki A, Toyokawa S, Inoue K, Miyoshi Y, Kobayashi Y (2016) Self-Reported Periodontitis and Incident Type 2 Diabetes among Male Workers from a 5-Year Follow-Up to MY Health Up Study. PLoS One 11 (4):e0153464. doi:10.1371/journal.pone.0153464

52. Myllymaki V, Saxlin T, Knuuttila M, Rajala U, Keinanen-Kiukaanniemi S, Anttila S, Ylostalo P (2018) Association between periodontal condition and the development of type 2 diabetes mellitus-Results from a 15-year follow-up study. Journal of clinical periodontology 45 (11):1276-1286. doi:10.1111/jcpe.13005

53. Winning L, Patterson CC, Neville CE, Kee F, Linden GJ (2017) Periodontitis and incident type 2 diabetes: a prospective cohort study. Journal of clinical periodontology 44 (3):266-274. doi:10.1111/jcpe.12691
