# Supplementary figures and images for: Epidemiologic relationship between periodontitis and type 2 diabetes mellitus
Source: BMC Oral Health. 2020 Jul 11;20:204. doi: 10.1186/s12903-020-01180-w (PMC7353775; doi:10.1186/s12903-020-01180-w)

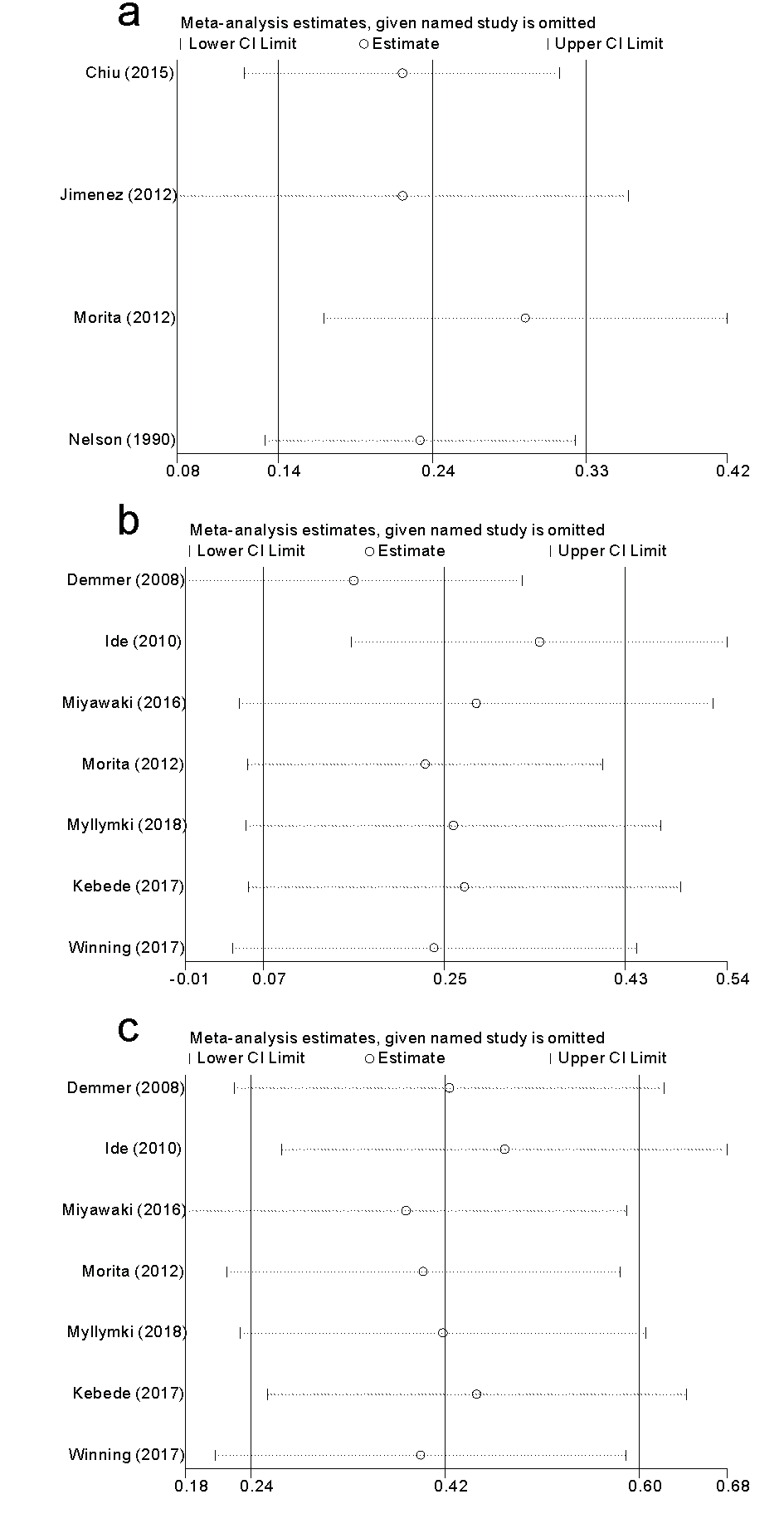

Supplement: Supplementary file 3 — Additional file 3. Appendix Fig. S2 Influence analyses of cohort studies (a) The impact of T2DM on periodontitis incidence (b) The impact of mild periodontitis on T2DM incidence (c) The impact of severe periodontitis on T2DM incidence [file 12903_2020_1180_MOESM3_ESM.tif]
